# TiN-Nanoparticulate-Reinforced ZrO_2_ for Electrical Discharge Machining

**DOI:** 10.3390/ma12172789

**Published:** 2019-08-30

**Authors:** Ana Lazar, Tomaž Kosmač, Janez Zavašnik, Anže Abram, Andraž Kocjan

**Affiliations:** 1 Department for Nanostructured Materials, Jožef Stefan Institute, 1000 Ljubljana, Slovenia; 2 Jožef Stefan International Postgraduate School, 1000 Ljubljana, Slovenia; 3 Max-Planck-Institut for Iron Research, Max-Planck Straße 1, 40237 Düsseldorf, Germany

**Keywords:** zirconia, titanium nitride, spark plasma sintering, nanocomposite, mechanical properties, electrical conductivity, electrical discharge machining

## Abstract

This study presents a fabrication route for an electrically conductive ZrO_2_–TiN ceramic nanocomposite with a nanoscale TiN phase occupying ≤30 vol% to improve the mechanical reinforcement of the zirconia matrix, and at the same time provide electrical conductivity to facilitate electro-discharge machining (EDM). The TiN nanoparticles were incorporated into a 3 mol% yttria-stabilized tetragonal zirconia (Y-TZP) powder, either by admixing a TiN nanopowder (MCP) or by using in-situ synthesis (ISS) via the forced hydrolysis of a titanyl sulphate aqueous solution and the direct nitriding of as-synthesized titania nanoparticles, followed by consolidation and rapid sintering in a spark plasma sintering (SPS) system. The initial phase composition and crystal structure of the as-synthesized powders and the sintered samples were characterized by transmission electron microscopy (TEM) and X-ray difraction (XRD). The influence of the different fabrication routes on the microstructural evolution, electrical and mechanical properties, and affinity for EDM were assessed using TEM, focused ion beam scanning electron microscopy (FIB-SEM, Vickers indentation, electrical conductivity measurements, and profilometry. The MCP synthesis route resulted in finer microstructures that are less prone to microstructural inhomogeneities; however, using the ISS route, it was possible to fabricate electrically conductive Y-TZP nanocomposites containing only 15 vol% of the TiN nanoparticulate phase. Both synthesis routes resulted in an increase of the fracture toughness with an increase of the TiN phase due to the nanoparticulate TiN reinforcement of the Y-TZP ceramic matrix via crack-bridging toughening mechanisms. As both synthesis routes yielded Y-TZP nanocomposites capable of successful EDM machining at a TiN content of ≥30 vol% for the MCP and ≥ 15 vol% TiN for the ISS, a possible mechanism was developed based on the microstructure evolution and grain growth.

## 1. Introduction

Yttria-stabilized tetragonal zirconia (Y-TZP) is a well-known engineering ceramic and is, due to its high strength, fracture toughness [1], and wear resistance, used in a variety of structural and functional applications, e.g., the machining and forming of metals, engine parts, radio-frequency heating susceptors, metrology components [2], as well as biomedical prosthesis [3] and dental restorations [4]. Owing to its useful mechanical properties, Y-TZP offers a promising ceramic matrix for electrically conductive (EC) composites with complex geometries that can be used for electrodes, sensors, or precision parts in micromechanics and electronic devices. However, in order to achieve the necessary complexity of the sintered, hard Y-TZP ceramics, especially for large production batches, expensive conventional machining with diamond tools is employed.

An alternative that can be used to overcome this problem is to use die sinking electrical discharge machining (EDM) or wire electrical discharge machining (WEDM) processes, if the electrical conductivity of a hard ceramic composite material is sufficient (>1 S·m^−1^) [5]. EDM is a subtractive manufacturing process for achieving complex shapes that is commonly used for machining hard alloys. EDM is effective in the removal of hard material via intensive thermal erosion, which may affect the surface and mechanical integrity of the material. The surface characteristics and surface properties of alloys can be improved, however, by post-processing steps such as grinding and etching-grinding, or when thick coatings are deposited on the surface to be machined, as was recently shown by Mandal et al. and Prakash et al., respectively [6,7].

The EDM of Y-TZP ceramic composites tends to be even more challenging due to the high electrical resistivity, high hardness, and brittle nature of ceramics. The EDM ability to a large extent depends on the electrical conductivity (EC) of the composite, while the EC of the composite is connected with the intrinsic EC of the phases and especially with the electrically conductive phase (ECP) distribution, aspect ratio, and the type of composite (nano–nano, nano–micro, micro–micro) [8]. Besides introducing EC to the insulating ceramic matrix, ECP in appropriate amounts can also contribute to an increased thermal conductivity, hardness, strength, and toughness [9,10,11]. However, successful fabrication usually requires high sintering temperatures to attain fully dense, electrically conductive Y-TZP ceramic composites, owing to the limited affinity for solid-state sintering and/or the pinning of the grain boundaries of the ECP.

Up to now, several ECP-containing Y-TZP-based composites that can potentially be machined with EDM have been prepared. To provide the necessary EC, the ECP can consist of various different materials; these can be transition-metal nitrides (TiN) [12,13,14,15,16,17], carbides (WC, TiC, ZrC, SiC) [15,18], carbonitrides (TiCN) [15], borides (TiB_2_) [15,19], or carbon nanofillers, such as carbon nanotubes (CNTs) [20], graphene [21], or even cellulose nanofibers that transform in-situ during sintering into the 3D network of few layers of graphene [22]. The machinability of Y-TZP-based ceramics with EDM as opposed to that of metals, is strongly related to the various factors determining the ECP-containing bulk, such as the ECP properties and microstructural homogeneity, as was shown by Gommeringer et al. [23]. Lauwers et al. demonstrated that WC and TiC were shown to be the most promising ECP used for making Y-TZP-based ceramics EC and processed with EDM [24].

When dealing with coarse-grained powders or inhomogeneous distributions, the content of the ECP in terms of the intrinsic EC can be up to 50 vol% so as to allow EDM [15]. The large amount of the introduced EC phase, however, can result in inhomogeneities that can negatively affect the mechanical properties and the reliability of the component [16]. One of the possible ways to lower the ECP content while achieving the necessary matrix percolation is to keep the ECP particles in the nanoscale range. However, nanopowders are prone to agglomeration and are difficult to homogeneously disperse in the Y-TZP matrix. Their high surface-to-volume ratio leads to an increased sintering activity, promoting grain growth during the conventional sintering process.

The nanoparticle (NP) agglomeration was shown to be successfully mitigated during in-situ synthesis (ISS) by the precipitation of titania nanoparticles on the host Y-TZP particles, as was shown in the Si_3_N_4_–TiN [25] and Al_2_O_3_–TiN [11] systems. In this way, TiN can be considered as a promising ECP candidate, even if it was inferior to TiC and WC when the conventional processing of coarse-grained powders were employed [26]. The NP growth (and phase separation) during sintering can be further prevented by using the spark plasma sintering (SPS) technique (also known as pulsed electric current sintering (PECS) or the field-assisted sintering technique (FAST)). SPS takes advantage of the electric field that can induce Joule heating (in the case of electrically conductive materials), increasing the diffusion rate at the grain boundaries and, thus, promoting densification via grain sliding. This means that enhanced densification of the sintered composites, preserving fine, nanoscale microstructures, can be obtained using SPS [27].

Several recent studies demonstrated an ability to prepare uniformly dense, fine-grained microstructures of either Y-TZP [28,29,30] or TiN [31,32,33] by SPS. However, there are only a handful of studies that have investigated the SPS of Y-TZP-TiN nanocomposites [34,35] that lead to dense composites possessing better mechanical and electrical properties than pure Y-TZP ceramics. The enhanced toughness (7.6 MPa·m^1/2^) and electrical conductivity (0.12 × 10^−6^ S·m^−1^) of composites containing 35 vol% TiN, sintered by FAST (1550 °C for 2 min, applying a pressure of 56 MPa), was reported by Vanmeensel et al. [36]. Hu et al. reports on fully dense composites containing nano-inclusions of 10–40 vol% TiN, sintered at 1200 °C, 20 min with an applied pressure of 80 MPa by SPS, resulting in a high electrical conductivity of 2724.4 S·m^−1^ and a rather modest fracture toughness of 3.44 MPa·m^1/2^ in the case of composites reinforced with 30 vol% TiN [34]. The hardness was moderately enhanced in the case of a TiN addition in the form of nanoparticles (15.4 GPa at 20 vol% TiN), while a micron-sized TiN reinforcement showed a slight increase in the hardness (13.75 GPa at 35 vol%) [14,34].

In the work presented here, we demonstrate that it is possible to fabricate electrically conductive ZrO_2_–TiN ceramic nanocomposites, with the TiN phase (≥15 vol%) remaining on the nanoscale, suitable for electro-discharge machining (EDM). The aim was to critically evaluate the mitigation of agglomeration during the composite processing, while the powder precursors, consolidated by SPS, were prepared by facilitating either the heterogeneous precipitation of titania nanoparticles on the surface of Y-TZP powder particles followed by a nitriding step in ammonia (ISS) or by mixing the Y-TZP and commercially available TiN nanopowder (MCP). Finally, the role of different powder-fabrication methods, varying the content of the nano TiN phase in the Y-TZP matrix, on the phase and microstructural evolution, fracture behaviour, electrical properties, and the ability of the as-prepared nanocomposites to be machined with EDM, were investigated.

## 2. Materials and Methods

### 2.1. Fabrication of the ZrO_2_–TiN Composite Powder

#### 2.1.1. Fabrication of the ZrO_2_–TiN Composite Powder via the Forced Hydrolysis of Titania Nanoparticles (ISS) and the Subsequent Direct Nitriding of the Synthesized Titania Nanoparticles

TiO_2_ nanoparticles were deposited on the surface of host 3 mol% yttria-stabilized tetragonal zirconia (Y-TZP) particles using the following procedure. Y-TZP powder (TZ-3Y-E, Tosoh, Japan) was dispersed in deionized water to obtain aqueous suspensions containing 10 wt% of solids loading. The suspension was homogenized by attrition milling at 600 rpm for 3 h using 3 mm zirconia balls. After 1 h of milling, citric acid as a dispersant was added to stabilize the zirconia suspension. After the slurry was milled, the aggregate break-up was further promoted with a Sonic vibra cell ultrasonic finger (30% amplitude, 10 s pulse, 5 s pause) for 20 min until full dispersion of the mixture. TiOSO_4_ (γ = 295 g/L; Cinkarna, Celje, Slovenia) was then added dropwise to the Y-TZP suspension during stirring. The precipitation of TiO(OH)_2_ was triggered by the dropwise addition of tetramethyl ammonium hydroxide (TMAH; Merck, Hohenbrunn, Germany) until a pH value of 4.7 was attained. The viscosity of the suspension increased, and the precipitates were separated from the mother solution using Buchner funnelling filtration. The filtrate was washed several times with deionized water, and at the end, also with anhydrous ethanol to eliminate the sulphate impurities. The cake was dried at 80 °C for 24 h and subsequently calcined at 600 °C for 1 h to transform the precipitated TiO_2_ precursor to crystalline TiO_2_ (anatase) NPs (as reported elsewhere [25]). The nitriding step for the as-modified Y-TZP powder was performed at 1000 °C for 3 h in flowing ammonia (150 mL/h) to transform the TiO_2_ to TiN.

#### 2.1.2. Fabrication of the ZrO_2_–TiN Composite Powder via Admixing of Commercial TiN Nanopowder to the Y-TZP Matrix (MCP)

Y-TZP (TZ-3Y-E, Tosoh, Japan) and nanosized TiN (PlasmaChem GmbH, Berlin, Germany) were attrition milled in ethanol at 600 rpm for 3 h using 3 mm zirconia milling balls. According to the supplier’s data, the average particle size of the TiN was 20 ± 5 nm with a specific surface area of 80 m^2^/g and 40 nm, with an area of 13–19 m^2^/g for the Y-TZP. After the milling, the suspension was oven-dried at 120 °C for 24 h, followed by sieving through an 80 µm mesh sieve.

### 2.2. Sintering

The sintering process was carried out with a SPS DC system (DR. SINTER^®^, model: SPS1050, producer: SPS Syntex Inc., Tokyo, Japan) using a graphite die with a 20 mm inner diameter. The die was surrounded by a porous graphite felt insulation to minimize the radiation heat losses. Then 3 g of composite powder were loaded into the die and sintered at 1300 °C, 5 min with an applied uni-axial pressure of 50 MPa. A heating rate of 100 K/min was used up to 1200 °C, afterwards, the final sintering temperature of 1300 °C was reached in 3 min to prevent overshooting of the set temperature.

### 2.3. EDM Processing

Ground and polished sintered samples were prepared from machined pieces using a die-sinking electrical discharge machine (IT Electronika 200M-E, Željko Volarič, Bovec, Slovenia) in dielectric oil. For all the machining experiments, a copper electrode of dimensions 5.17 mm × 5.17 mm was used. The EDM was programmed to machine for a time of 10 min. The following parameters were used: voltage 280 V, current 3.3 A, discharge duration 28 μs, and pause duration 80 μs.

### 2.4. Characterization

The density of the sintered samples was measured using Archimedes’ method of immersing the bulk into deionized water at 25 °C. The theoretical density was calculated using the density of TiN (5.22 g·cm^−3)^ and 3Y-TZP (6.05 g·cm^−3^).

The XRD spectra of the sintered samples used for the phase composition and the Rietveld analysis for the crystallite size determination of the TiN were examined using an X’Pert PRO MPD X-ray diffractometer (PANalytical, Almelo, Netherlands) with Cu-Kα_1_ radiation at 45 kV and 40 mA with the following set-up: 2θ from 25° to 75°, step 0.034, time 100 s, with the X’Celerator detector fully opened (2.122°).

The microstructures of the sintered and EDM-ed composites in top-down and cross-section views were analysed with a scanning electron microscope coupled with a focused ion beam (SEM-FIB, Helios Nanolab 650, FEI, Hillsboro, OR, USA). Before the analysis, a 0.5 µm-thick layer of platinum film was deposited onto the area of interest, using an ion-beam-assisted gas-injection system at 30 kV and 0.43 nA to preserve the very thin surface and to mitigate the curtaining effect. FIB trenches for the cross-section observation were cut with a Ga-ion beam at 30 kV and 65 nA and finalized by ion polishing at 30 kV and 21 nA. The cross-sections were observed in situ at an angle of 52° using electrons at 5 kV and 80 pA. The chemical compositions of the samples were analysed utilizing an energy-dispersive X-ray spectrometer (EDXS, X-MaX SDD, Oxford instruments, Abingdon, UK) at 15 kV and 0.8 nA.

The size of the NPs, their crystal structure and morphology were analysed using a transmission electron microscope (TEM; JEM-2010F Jeol, Tokyo, Japan). The fine powders were dispersed in EtOH_(abs)_ and sonicated in an ultrasonic bath, and then transferred to commercially available Cu-supported amorphous carbon grids. The sintered ceramic samples were prepared using a conventional sample preparation procedure combined with mechanical and Ar^+^-ion thinning [37] and analysed using a S-TEM (2200FS, Jeol) operating at 200 kV and additionally equipped with a large-angle SDD-EDS detector (Jeol).

The Vickers hardness (HV_10_) was measured using a hardness tester (Innovatest, Nexus 7500, Maastricht, Netherlands) with an indentation load of 98.1 N for 10 s. The fracture toughness, K_ifr_, based on a crack-length measurement of the radial crack pattern produced by Vickers HV_10_ indentations, was calculated according to the formula of Niihara et al. [38].

The electrical resistivity of the sintered materials was measured with a DC Multimeter-3457A-testing machine (HP, Palo Alto, CA, USA) using Van der Pauw at room temperature (25 °C) with a direct current. The samples were ground and polished to plan parallel.

The surface roughness of the die sunk surfaces was measured in terms of R_a_ using a 2D surface profilometer (Talysurf, Series 2, Taylor-Hobson, Leicester, UK), each value being the mean of three runs over a travelled length of 5 mm, spaced 0.1 mm apart.

## 3. Results and Discussion

### 3.1. Fabrication of ZrO_2_–TiN Composite Nanopowders

**Figure 1:**
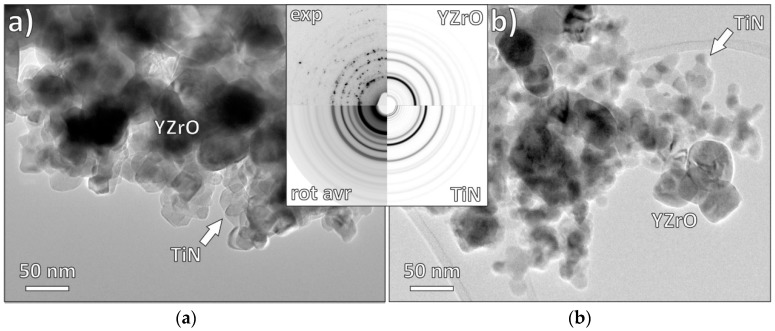
TEM micrographs of (**a**) TiN-coated Y-TZP powder particles (15ISS) and (**b**) admixing of commercial TiN nanopowder with Y-TZP (15MCP). In both cases, the SAEDP (inset; exp = experimental pattern, rot avr = rotational average of experimental pattern, YZrO, and TiN are calculated simulations) corresponds to a mixture of pure tetragonal YZrO and cubic TiN, without intermediates.

ZrO_2_–TiN composite nanopowders with different nominal starting compositions, containing 7.5, 15 and 30 vol% TiN, were prepared either by in-situ synthesis (ISS) (precipitation by forced hydrolysis) or the mixing of commercial powders (MCPs). In Figure 1, the representative TEM morphologies of ZrO_2_–TiN powders containing 15 vol% TiN are shown. TiN nanoparticles with an average size of 13 nm reside on the surface of the Y-TZP as a result of a successful precipitation via a forced hydrolysis that was followed by nitriding (Figure 1a); the selected-area electron diffraction pattern (SAEDP, Figure 1 inset) analysis confirmed the presence of a mixture of pure Y-TZP and TiN crystallites with random orientations. In Figure 1b, a TEM micrograph of the composite powder prepared by the mixing of commercial TiN nanopowder together with Y-TZP powder is presented. The average measured TiN particle size was 18 nm, which is in good agreement with the specifications provided by the manufacturer. As the average TiN crystallite size is the main difference between both samples, it is worth mentioning that, in the case of commercial powder, the size distribution of the TiN crystallites is narrow, in the range of 16–20 nm (average 18 nm), while in the case of in-situ precipitation, the size distribution is bimodal, with minor TiN crystallites in the size range 5–10 nm and the majority in the size range 10–15 nm (average 13 nm). As a result, we can expect a much better, homogenous distribution in the case of ISS, while in the case of MCP, the TiN nanoparticles are expected to form distinct agglomerated clusters.

### 3.2. Sintering and Phase Composition

SPS of the composite powders at 1300 °C for 5 min with an applied uniaxial pressure of 50 MPa resulted in the complete densification of the sintered nanocomposites (>97%), irrespective of the powder-preparation procedure used. For comparison, Hu et al. sintered fully dense Y-TZP nanocomposites containing 10–40 vol% TiN with a mean grain size of 55–91 nm at an even lower SPS temperature, i.e., 1200 °C; however, they used a prolonged dwell time of 20 min and an applied pressure of 80 MPa [34]. In the present study, the relative density of the spark-plasma (SP) sintered composites decreased with an increasing content of TiN (Table 1). However, the bulk densities of the sintered ISS samples, compared to the MCP samples, were slightly lower because of the inhomogeneities present (agglomerated TiN), which is much more pronounced at higher TiN contents.

**Figure 2:**
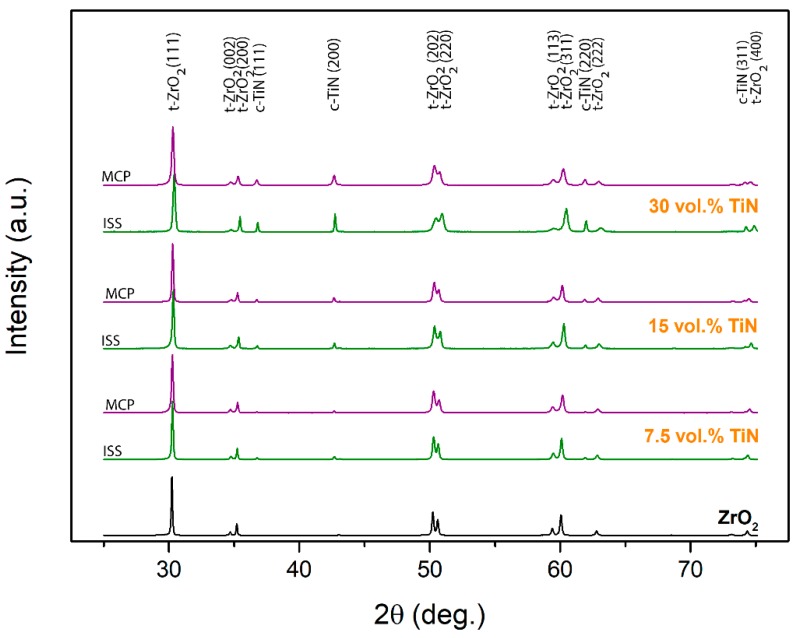
XRD patterns of SP-sintered nanocomposites for various powder compositions prepared by ISS (green) and MCP (purple) fabrication routes.

In Figure 2, the XRD patterns of SP-sintered composites differing in TiN content prepared by ISS and MCP are shown. For comparison, pure Y-TZP SP-sintered under the same conditions is also presented. In all the composites, the 3 mol% yttria-doped tetragonal ZrO_2_ and the cubic TiN (osbornite) phase were the only crystalline phases present. No monoclinic ZrO_2_ nor titania residues were observed, which, in the case of ISS composite powder, indicated the complete nitriding process for the precipitated titania NPs, leading to TiN [25]. As expected, the peak intensity of the TiN increased with the increasing content of TiN, regardless of the fabrication route used for the composite powders.

The Rietveld analysis was conducted based on all the patterns and the results are presented in Table 1. The calculated content of TiN in the composites deviated slightly from the targeted amount and was, except for the 7.5 vol% TiN ISS sample, lower than the set values. The calculated average crystallite sizes of the TiN in the SP-sintered samples were increasing with the amount of the TiN phase for the ISS fabrication route, i.e., 55, 70, and 98 nm for the 7.5, 15, and 30 vol% TiN, respectively. However, in the case of MCP, the TiN average crystallite size remained practically unchanged and was around 65 nm. The largest calculated crystallite size agrees well with the much narrower and more intensive (111) peak of TiN, in the case of ISS (Figure 2).

### 3.3. Microstructural Evolution

**Figure 3:**
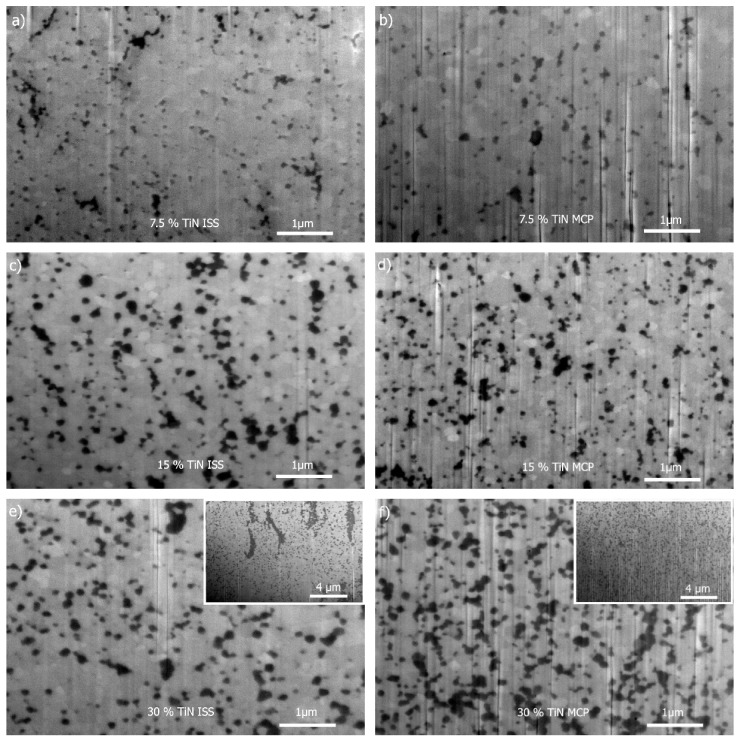
Back-scattered SEM micrographs of SP-sintered samples for various powder compositions prepared by the ISS (**a**,**c**,**e**) and MCP (**b**,**d**,**f**) fabrication routes.

The microstructures of the SP-sintered ZrO_2_–TiN composites with various powder compositions prepared by the ISS (a,c,e) and MCP (b,d,f) fabrication routes are presented in Figure 3; the cross-sections (wells) were prepared by FIB from the bulk.

In the composites containing 7.5 and 15 vol% of TiN, the TiN grains (dark coloured) were homogeneously dispersed in the Y-TZP matrix (bright coloured grains) irrespective of the fabrication route employed. As expected, increasing the content of TiN from 15 to 30 vol% resulted in an obvious increase in the TiN dark-coloured grains that were, in the case of MCP (Figure 3f), still several relatively homogeneously distributed in the microstructure that also contained distinct agglomerates of several grains in contact. It seemed that the size of the TiN grains remained similar, irrespective of the TiN content in the case of MCP, which corroborated well with the calculated crystallite size (Table 1). On the other hand, in the case of ISS, increasing the TiN content from 15 to 30 vol% did not result in a systematic increase on the micro-scale, as observed in Figure 3d,f which exhibited similar microstructures (Figure 3c,e). However, there were regions containing extensive amounts of segregated TiN grains, forming a channel-like agglomeration (inset of Figure 3e), which could be ascribed to the homogeneous instead of heterogeneous type of precipitation of the titania nanoparticle precursor in the titanyl sulphate aqueous solution with a higher TiOSO_4_ concentration and/or agglomeration/flocculation of the ZrO_2_ particles, resulting in an inhomogeneous TiN distribution in the composite powder. In contrast to the MCP, the grain size in the SP-sintered ISS-prepared composites (Figure 3a,c,e) was gradually increased, as was also the case for the calculated crystallite sizes (Table 1). The latter could be ascribed to the higher reactivity of the TiN particles prepared by ISS (forced hydrolysis), owing to the higher surface energy and/or the amorphous phase that might have provoked rotation and ordered coalescing during the rapid sintering in the SPS equipment. Similar coalescence processes were already observed in the case of the pure Y-TZP [39] and ZrO_2_–TiN (>30 vol%) systems. Hu et al. speculated that the reason lies in the high volume fraction of TiN or the difficulty in achieving a complete particle deagglomeration by ball milling [34].

**Figure 4:**
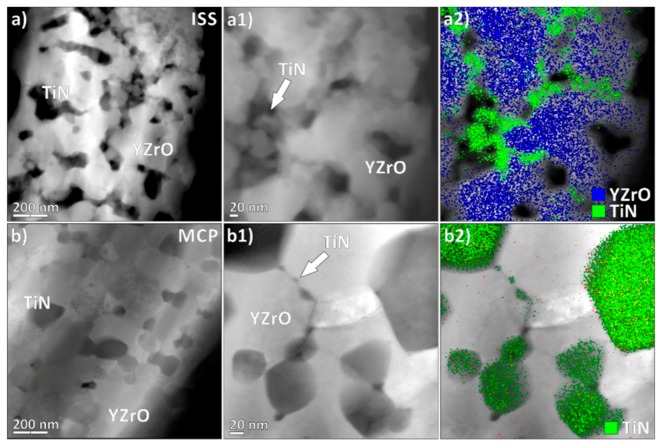
HAADF-STEM micrographs of sintered (**a**,**a1**) ISS and (**b**,**b1**) MCP ZrO_2_–TiN ceramic, with corresponding EDS overlay maps (**a2**,**b2**) of Zr L and Ti K, interpreted as Y-TZP and TiN, respectively.

The TEM investigation of the ZrO_2_–TiN sintered MCP composites revealed large TiN grains accompanied by numerous, up to 10 nm-sized, TiN particles that are concentrated on the grain boundaries. In the case of ISS, the TiN phase is continuously distributed at grain boundaries, also forming distinct concentrated clusters, as seen in the Figure 4a) series, while in the case of MCP, the TiN phase has a strong bimodal size distribution, consisting of large, well-defined grains, mainly on the triple-junction grain boundaries, accompanied by nano-sized TiN particles scattered on the low-index grain boundaries. EDS mapping in the High-Angle Annular Dark-Field Scanning Transmission Electron Microscopy (HAADF-STEM) was used to reveal the spatial distribution of both clearly separated phases, positioned preferentially on the grain boundaries (in Figure 4, Ti K-lines were used as a marker for the TiN phase and the Zr L-line family was used as a marker for the Y-TZP phase). The obtained results are additional support for the relative-density calculations presented in Table 1, where MCP shows a generally denser microstructure, mainly related to the absence of TiN agglomeration (as seen in ISS) and the relatively dense distribution of nano-sized TiN grains on the grain boundaries (GBs). Normally, such positioning on the GBs is undesirable, as it can decrease the mechanical properties of the material, but in our case, it can significantly improve the electric conductivity. As SPS utilizes Joule heating provided by an electric current via the conductive lining and through the sample, the conductivity provided by the TiN NPs is an important factor, even at the SPS synthesis stage, as the TiN NPs can serve as local hot spots.

### 3.4. Mechanical Properties

**Figure 5:**
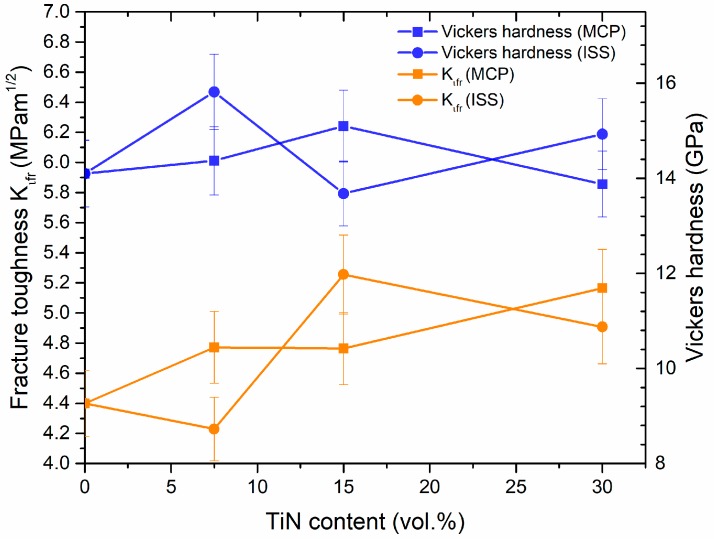
Indentation toughness and Vickers hardness as a function of the TiN content of the SP-sintered (1300 °C, 5 min, 50 MPa) composites fabricated by the ISS and MCP routes.

The SP-sintered ZrO_2_–TiN nanocomposites prepared by both routes were tested for Vickers hardness and fracture toughness, and the results are presented in Figure 5.

It was observed that all the composites had slightly higher hardness values compared to the SP-sintered pure 3Y-TZP sample (14.1 GPa), varying between 14 and 16 GPa, on account of the fine microstructures produced by the SPS, as well as the presence of TiN nanoparticles at the grain boundaries of the Y-TZP acting as pinning agents that prevent grain growth. The differences in the hardness for the samples with a larger amount of TiN, however, were statistically insignificant.

Figure 5 (orange lines) shows a plot of indentation toughness as a function of TiN content. All the sintered composites, regardless of the preparation route, had higher toughness values than the pure 3Y-TZP (4.4 MPa·m^1/2^). Similar to the hardness values, the K_ifr_ values, the differences with respect to the samples with a larger amount of TiN, however, were statistically insignificant, with the highest values reaching 5.2 MPa·m^1/2^. Vleugels et al. reported even higher fracture–toughness values for similar composites, but they were using a mixture of the monoclinic and tetragonal (3Y-TZP) forms of ZrO_2,_ resulting in a lower net yttrium (2.8 mol%) content that facilitates a tougher Y-TZP matrix (8.2 MPa·m^1/2^) [15].

**Figure 6:**
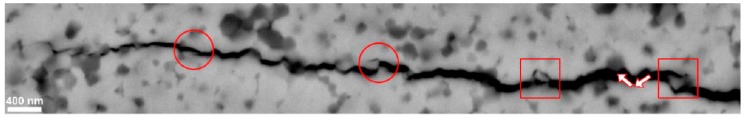
SEM micrograph showing an arrested crack propagated from the Vickers indent. Circles, squares, and arrows indicate the crack branching, crack bridging, and crack deflection, respectively.

The increase in the toughness can be attributed to the addition of nano-sized TiN particles into the Y-TZP matrix acting as inclusions and provoking bridging mechanisms when the crack is being propagated (or arrested during indentation). In Figure 6, all three bridging toughening mechanisms are visible, i.e., crack deflection, crack bridging, and crack branching were observed in the end part of the crack that propagated from the indent.

**Figure 7:**
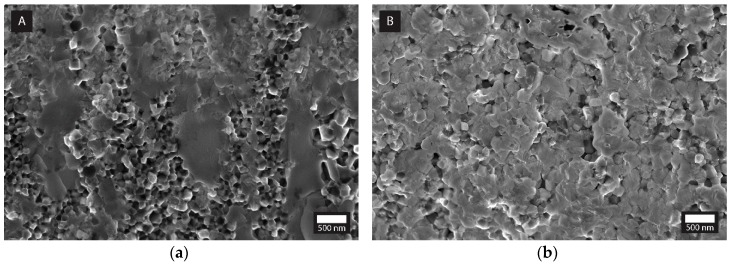
SEM micrographs of fracture cross-sections for ZrO_2_-30TiN nanocomposites (**a**) ISS, (**b**) MCP.

A generally slightly higher indentation toughness observed in the case of the MCP fabricated nanocomposites, especially for the 7.5 and 30 vol% TiN additions, (Figure 5), could be ascribed to the more homogeneous microstructures obtained, as well as to the smaller average TiN crystallite sizes (Table 1) acting as inclusions to more effectively pin the grain boundaries, besides interacting with the propagating crack (Figure 6). In Figure 7, the fractured surfaces of the SP-sintered samples containing 30 vol% TiN prepared by both fabrication procedures are shown. Indeed, the more efficient grain-boundary pinning in the MCP sample can be assumed from the fact that the fractured surface of the ISS sample exhibits predominantly intergranular fractures and slightly smaller Y-TZP grains, as compared to the MCP sample, where there is a substantial amount of transgranular fracture. Larger Y-TZP grains are known to exhibit a higher stress-induced tetragonal-to-monoclinic toughening [40]. The same observations relating to MCP-prepared composites were already reported by other authors [34,35,41].

### 3.5. Electrical Properties and Electro-Discharge Machining

The size, shape, and homogeneous distribution of the ECP in the insulating matrix, ECP-matrix interactions, and processing technique are key factors in defining the percolation concentration [8]. In the present study, the nanocomposites prepared by both fabrication routes exhibited relatively homogeneously distributed TiN particulates ECP (Figure 3) and, thus, it was expected that the SP-sintered composites containing nano-sized TiN grains, especially those prepared by ISS synthesis [11,42], will exhibit a higher EC at lower contents of TiN phase compared with the composites containing micron-sized inclusions of TiN phase [14,15,16,17]. The results of the EC measurements are presented in Table 2. The amount of 7.5 vol% TiN phase in the Y-TZP matrix, even if in a nanoscale particulate form, was still insufficient to achieve percolation. Increasing the TiN content to 15 vol% (~13 vol% calculated; Table 2) turned out to be a threshold value for achieving percolation of the SP-sintered ZrO_2_–TiN nanocomposites, since the ISS fabrication route was just above percolation, while the MCP was still below. A further increase of the TiN to 30 vol%, corresponding to the calculated values of 21.6 and 24.1 vol% for ISS and MCP, respectively, provided a very large EC (Table 2). The EC of 30ISS was more than two times higher than the 30MCP. This could be the consequence of the formed continuous TiN phase, which provides a conductive path in the ISS composites (Figure 3e and Figure 4a2). A similar dependence of the preparation method on EC was reported by Gao et al., who investigated TiN–Al_2_O_3_ nanocomposites [42].

The onset of percolation achieved at about 15 vol% of the conductive phase was in line with the work of Gao et al. and Li et al., who investigated TiN–Al_2_O_3_ nanocomposites prepared by in situ coprecipitation and the precipitation method of a TiO_2_–αAl_2_O_3_ precursor, further nitriding in ammonia gas to obtain TiN–Al_2_O_3_ and consolidation by hot pressing for 60 min at 1400–1650 °C with a pressure of 30 MPa under a N_2_ atmosphere. The TiN and α-Al_2_O_3_ particle sizes are comparable with the results reported here, i.e., between 40 and 50 nm and approximately 300 nm, respectively [11,42].

**Figure 8:**
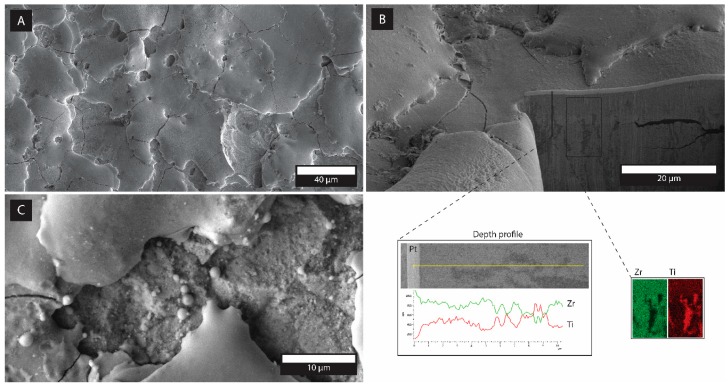
Back-scattered SEM micrographs of EDM-machined 30 vol% TiN–ZrO_2__ISS (**a**) surfaces (**b**) surface at higher magnification indicating the material–removal mechanisms, and (**c**) subsurface with an inset of the EDS of the area containing the TiN agglomerate.

In accordance with the literature [5], composites that attained a conductivity of at least 0.01 S∙cm^−1^ could be machined with EDM. Nevertheless, the surface quality of the machined composites varies a great deal with the EC and machining parameters [26]. The results of the investigated machined composites in terms of the material–removal rates and surface roughness are presented in Table 2, while the microstructures of the EDM-machined surfaces are represented in Figure 8. The surface roughness of the machined composites are comparable with those machined with standard EDM parameters (8 A, duty factor 11%, pulse duration 1.6 µs). The achieved surface finishes in the range 2–4 µm and the rather modest material–removal rate was acceptable in terms of the mild machining parameters. Applying more severe EDM parameters could result in higher volumetric material–removal rates as in the case of Put et al. and Pitman [26,43]. The machinability in terms of the material–removal rate, although modest, was better in the case of the ISS-fabricated composites, given their much more pronounced EC (Table 2).

The surface microstructures after the EDM process were, as expected, similar for both fabrication routes, as were the measured roughnesses (Table 2). The typical microstructure of the EDM-machined surface and the subsurface of the 30ISS composite is presented in Figure 8. The edge of the pit was sharp (not shown), while the bottom surface exhibited an orange-peel-like surface finish, interlaced with propagating surface cracks (Figure 8a), which was indicative of the intense melting and re-solidification processes of the material during the subtractive EDM process. In the higher-magnification images (Figure 8c), spherical precipitates and pore channels, where the material was possibly drained out from the bulk, are visible. Nevertheless, the (sub)surface layer was relatively coherent with no delamination or phase segregation. Surface cracks were, however, propagating into the vicinity of the near subsurface, but were oriented parallel to the EDM surface (Figure 8b). In the inset of Figure 8b, the results of the EDS mapping, besides showing a homogeneously dispersed TiN phase (Ti in red) within the ISS sample, also confirmed the presence of the channel-like agglomeration of TiN phase composed of TiN nanoparticles (also observed in the inset of Figure 3e, Figure 4a1,a2 and Figure 7a) being positioned perpendicular to the disk surface. The latter might be the reason for the substantial increase in the electrical conductivity of the ISS samples compared to the MCP samples that contained ≥15 vol% of TiN phase, which was obviously high enough to achieve the EC required for EDM.

## 4. Conclusions

We have demonstrated that it is possible to fabricate mechanically reinforced and electrically conductive ZrO_2_–TiN ceramic nanocomposites with the TiN phase (≤30 vol%) remaining on the nanoscale that are suitable for electro-discharge machining (EDM), either admixing a commercially available TiN nanopowder (MCP) or by using an in situ forced hydrolysis of titanium oxysulphate solution and the direct nitriding of synthesized titania nanoparticles (ISS). The following conclusions can be drawn:

- For both syntheses approaches, the full densification, using a spark plasma sintering (SPS) system, was achieved at 1300 °C and 50 MPa for 5 min.
- Using the MCP route led to slightly coarser microstructures, being less prone to microstructural inhomogeneities, leading to an increase in the indentation toughness due to the nanoparticulate TiN reinforcement of the Y-TZP ceramic matrix because of the crack-bridging toughening mechanisms.
- Using the ISS route, it was possible to fabricate electrically conductive Y-TZP nanocomposites containing only 15 vol% of the TiN nanoparticulate phase, presumably due to the formation/presence of the channel-like agglomeration of the TiN phase.
- Both routes yielded Y-TZP nanocomposites that were successfully machined by EDM when the TiN content was 15 and 30 vol% in the case of ISS and MCP, respectively.

## Figures and Tables

**Table 1:** Relative density and TiN crystal size as a function of composition, as determined by the Rietveld analysis.

Composition (vol%)			Relative Density (%)		TiN Crystal Size (nm) (Goodness of fit—χ^2^)	
Targeted	Calculated					
	ISS	MCP	ISS	MCP	ISS	MCP
7.5	9.8	4.9	98.3	99.9	55 (2.23)	66 (2.69)
15	12.8	13.0	98.0	99.5	70 (3.06)	64 (2.39)
30	21.6	24.1	97.6	98.3	98 (2.76)	64 (2.12)

**Table 2:** Electrical conductivity, material removal rates and surface roughness of composites machined by die sinking electrical discharge machining.

Composition (vol% TiN)	Electrical Conductivity (S·m^−1^)		Material Removal Rate MRR (mm^3^min^−1^)		Surface Roughness, Ra (µm)	
	ISS	MCP	ISS	MCP	ISS	MCP
7.5	<1 × 10^−4^	<1 × 10^−4^	-	-	-	-
15	2	<1 × 10^−4^	0.002	-	2.6	-
30	7.0 × 10^4^	2.9 × 10^4^	0.011	0.005	4.1	4.5
